# Improved false negative rate of axillary status using sentinel lymph node biopsy and ultrasound-suspicious lymph node sampling in patients with early breast cancer

**DOI:** 10.1186/s12885-015-1331-9

**Published:** 2015-05-09

**Authors:** Yulong Wang, Haiyan Dong, Hongyan Wu, Li Zhang, Kai Yuan, Hongqiang Chen, Mingwen Jiao, Rongzhan Fu

**Affiliations:** 1 Department of Breast and Thyroid Surgery, Subsidiary Qianfoshan Hospital of Shandong Universityf, Jinan, 250014 Shandong Province China; 2 Department of Endoscopy, Subsidiary Qianfoshan Hospital of Shandong University, Jinan, 250014 Shandong Province China; 3 Department of Health Management Center, Subsidiary Qianfoshan Hospital of Shandong University, Jinan, 250014 Shandong Province China; 4 Department of Ultrasound, Subsidiary Qianfoshan Hospital of Shandong University, Jinan, 250014 Shandong Province China

**Keywords:** Breast cancer, Sentinel lymph node biopsy, Axillary lymph nodes dissection, Ultrasound

## Abstract

**Background:**

The false negative rate of sentinel lymph node biopsy (SLNB) is 5-10%, and results in improper patient management. The study was to assess the value of ultrasound-suspicious axillary lymph node biopsy (USALNB) in patients with early breast cancer, and to compare SLNB combined with USALNB (SLNB + USALNB) with SLNB alone.

**Methods:**

From January 2010 to July 2013, 216 patients with early breast cancer were enrolled consecutively at the Department of Breast and Thyroid Surgery, Qianfoshan Hospital, Shandong University. All patients underwent wire localization of the suspicious node by color Doppler ultrasonography, followed by SLNB 2–3 hours later, suspicious node lymphadenectomy, and level ≥ II axillary dissection (as the gold standard). The predictive values of node status between SLNB + USALNB and SLNB alone were compared.

**Results:**

The success rate of SLNB was 99.1% (214/216). After axillary dissection, 71 patients were confirmed with axillary lymph node metastases by pathological examinations. Eight false negatives were observed using SLNB alone, resulting in sensitivity of 88.7%, specificity of 100%, false negative rate of 11.3%, and false positive rate of 0% in predicting the axillary node status. SLNB + USALNB resulted in sensitivity of 97.2%, specificity of 100%, false negative rate of 2.8%, and false positive rate of 0%. The false negative rate of SLNB + USALNB was significantly different from that of SLNB alone (P = 0.031).

**Conclusions:**

SLNB + USALNB seems to be a low-risk procedure that might be useful in reducing the false negative rate of SLNB, improving the accuracy of axillary nodes evaluation in early breast cancer.

## Background

The presence or absence of axillary lymph node involvement represents one of the most important prognostic indicators of long-term patient outcome for breast cancer. In that same regard, accurate axillary staging is extremely important for guiding the surgical management and for directing the appropriate selection of adjuvant therapies for breast cancer. Sentinel lymph node biopsy (SLNB) is usually performed first because it is less morbid than axillary lymph node dissection (ALND). Therefore, patients with negative SLNB may avoid unnecessary ALND and its complications such as upper extremity edema and shoulder joint movement disorders [1]. However, the SLNB technique is associated with a false negative rate of 5 to 10% [2-4]. This high false negative rate is of clinical concern, and thus new approaches to axillary lymph node staging are clearly needed to address this issue.

Identification and sampling of suspicious axillary lymph nodes (SALN) using ultrasound has been proposed in order to improve axillary staging without the necessity to perform an ALND. Indeed, a number of recent studies used ultrasound to identify SALN and to sample them, either using fine needle aspiration (FNA) and/or core needle biopsy, and showed relatively good predictive value for axillary status [5-7]. However, most of these studies tested the use of ultrasound-guided SALN biopsy (USALNB) instead of SLNB, and the use of USALNB alone is associated with highly variable false positive rates [5] that may impair its use in a clinical setting. Nevertheless, the use of ultrasound to detect the SALN was shown to reduce the reoperation rate [8,9].

It is a well-accepted concept that the lymphatic spread of tumor cells from the primary breast cancer site occurs through defined regional lymphatic pathways leading to the axillary region and to other regional lymphatic chains. Therefore, tumor cells from the primary breast cancer site will first be recognized within a defined lymph node or defined group of lymph nodes which we designate as the sentinel lymph nodes (SLNs) [10]. The ability to recognize the presence of tumor cell within any given lymph node is the basis of accurate axillary staging for breast cancer. While it is intuitive that the early involvement of any given lymph node by tumor cells may not result in any easily recognizable alternations in the morphologic characteristics of a lymph node on ultrasound, and that it may require a greater degree of lymph node involvement to become sonographically apparent, the use of ultrasound for axillary staging in breast cancer reasonably well-established [11]. The use of ultrasound for axillary staging in breast cancer could be thought of as complementary to SLNB, as SLNB is not without its own issues as related to false negativity rates [4].

Therefore, in the present study, we evaluated a new approach for axillary staging using both SLNB and USALNB by wire localization. In our study design, we attempted to evaluate this new approach for predicting the axillary status by comparing SLNB alone technique to that of a combined SLNB + USALNB technique, and utilized concomitant ALND as the gold standard.

## Methods

###  Patients

Between January 2010 and July 2013, 216 consecutive patients were enrolled at the Department of Breast and Thyroid Surgery, Qianfoshan Hospital, Shandong University. Inclusion criteria were: 1) women with clinical stage I or II breast cancer according to the AJCC TNM version 6.0 [12]; 2) a single primary breast tumor; 3) no distant metastases; and 4) no history of surgery or radiotherapy on the same side as the cancer. Exclusion criteria were: 1) neoadjuvant chemotherapy; 2) neoadjuvant endocrine therapy; 3) local resection surgery performed before axillary staging; or 4) radiotherapy on the same side as the cancer. Diagnosis was confirmed by the pathological examination of percutaneous breast biopsies.

The study was approved by the Medical Ethics Committee of the Qianfoshan Hospital affiliated to Shandong University (20110133), and written informed consent was obtained from each participant.

### Study design

This was a cohort study aiming to determine the predictive value of the axillary status in early breast cancer using SLNB alone and SLNB+ USALNB compared with axillary dissection as the gold standard. All patients underwent, in order: ultrasound-guided wire localization of the suspicious lymph node, SLNB, USALNB, and ALND.

### Lymph node sampling

**Figure 1:**
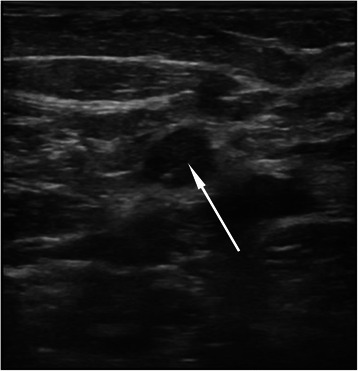
Ultrasound localization of suspicious lymph nodes 2 hours before SLNB. The red arrow shows the hook wire.

B-mode ultrasound (Logiq 9, GE Healthcare, Waukesha, WI, USA) was conducted to determine the axillary lymph node that was the most likely to arbor a breast cancer metastasis (Figure 1). Because there is no generally accepted definition of suspicious lymph nodes under ultrasound, a suspicious lymph node was defined in the present study as a lymph node >0.5 cm in diameter, a length/width ratio <1.7, absence of hilum, heterogeneous thickening of the cortex, and increased peripheral blood flow [13]. Lymph nodes showing these features were present in almost all patients. Therefore, after a careful examination of each lymph node, the most suspicious lymph node was selected for sampling. A hook wire (US Biopsy Breast Location Needle, Promex Technologies LLC, Franklin, IN, USA) was used to identify the one or two most suspicious lymph nodes under ultrasound guidance. If two lymph nodes were very close, the lymph nodes were identified with a single hook wire. On the other hand, if the lymph nodes were distant from each other or could not be observed in one ultrasonic range, two hook wires were used.

Two radiologists including one chief and one senior attending doctor were asked to identify the suspicious lymph nodes. Disagreements were discussed and resolved by consensus.

**Figure 2:**
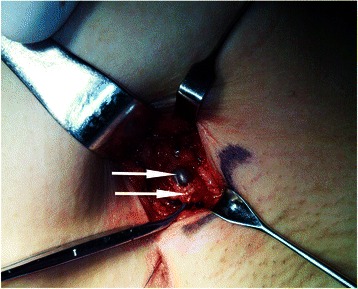
Identification of suspicious lymph node and SLN during surgery. The green arrow shows the methylene blue-dyed SLN, while the yellow arrow indicates the adjacent suspicious lymph node that was identified by preoperative ultrasound and wire localization. In this patient, final pathology confirmed that the SLN had no metastasis, while a metastasis was present in the suspicious lymph node.

SNLB was performed 2–3 hours after ultrasound. A subcutaneous injection of methylthioninium chloride (4 ml, 40 mg; Jiangsu Jumpcan Pharmaceutical Group Co., Ltd., China) was performed at the surface of the tumor. Methylene blue was used instead of isosulfan or isotopes because of the limited availability of these products. The skin and subcutaneous tissues were incised 10 min later, and skin flaps were isolated routinely. Adipose connective tissues were incised parallel to the outer edge of the pectoralis major muscle. After identification of the blue-stained lymphatic vessels, dissection of the tissues was performed along the lymphatic vessels to find and resect blue-stained lymph nodes. The resected blue-stained lymph nodes were labeled as the sentinel lymph nodes for pathological examinations (Figure 2).

Thereafter, the nodes identified using ultrasound were removed along the wire. During SLNB, the consistency between the nodes identified by SLNB and those identified using ultrasound was examined (Figure 2). The two methods were considered consistent when the nodes identified by ultrasounds were found to be stained blue and inconsistent when nodes other than the ones identified by ultrasound were stained blue. The ultrasound-suspicious lymph nodes were all removed with the hook wire, regardless of SLNB results. Lymph nodes identified by hook wires were considered as suspicious lymph nodes, while blue-stained ones were considered as SLNs.

Suspicious lymph nodes and SLNs were examined using frozen sections. Axillary lymph nodes may be divided according to the outer, rear, and inner sides of the pectoralis minor muscle groups, according to the conventional surgical grouping criteria of axillary lymph nodes. In all cases, a standard ALND of levels I/II or levels I/II/III was performed regardless of the results of SLNB and/or USALNB. Breast-conserving surgery or mastectomy was then performed, based on the decision reached by the patient following discussion with the surgeon. Adjuvant chemotherapy, radiotherapy, hormonal therapy, and biological therapy were administered post-operation according to each cancer, in a standard manner.

All lymph nodes sampled were subjected to standard pathological examination, including hematoxylin & eosin (H&E) staining, and estrogen receptor, progesterone receptor and HER2 immunohistochemistry. Cytokeratins were not evaluated in the lymph nodes due to the limited amount of available tissues. H&E staining were considered as the gold standard to assess sensitivity and false negative rate.

Pathologists were blind regarding the origin of the lymph nodes. All researchers were blind to the pathological results.

### Follow-up

All patients were followed up after treatments were completed. Follow-up was performed every 6 months by plain X-ray of the chest, bone scanning, ultrasound examination of the liver, thoracic wall and breasts, and axillary ultrasound examination, according to the NCCN guidelines.

### Statistical analysis

Data are presented as mean ± standard deviation (SD), absolute numbers and percentages. Variables within the contingency tables were analyzed using the χ^2^ or the Fisher exact test, as appropriate. SPSS 16.0 (SPSS Inc., Chicago, IL, USA) was used for statistical analysis. P-values <0.05 were considered statistically significant.

## Results

### Patients’ characteristics

Age ranged from 26 to 68 years old. All patients were without clinically palpable nodes before surgery. There were 76 patients with clinical stage I breast cancer and 140 patients with clinical stage II breast cancer (according to the AJCC TNM, 6th edition). Tumor was found in the upper outer quadrant in 96 cases, in the lower outer quadrant in 54 cases, in the upper inner quadrant in 12 cases, in the lower inner quadrant in 10 cases, and in the central quadrant in 44 cases. Of these cases, 196 underwent modified radical mastectomy, and 20 underwent breast-conserving surgery. Most tumors were invasive ductal carcinomas (85.2%). Tumor grade was I in 10.2% of cases, II in 83.3% and III in 6.5%. Tumors were hormone receptor-positive in 66.2% of cases, HER2-positive in 19.4%, and 68.1% showed a Ki-67 index >14% (Table 1).

**Table 1:** **Patients' demographic and clinical characteristics**

Characteristics	Value
Age	
Mean ± SD (years)	52.1 ± 10.3
Range	22-66
≤50 years	99 (45.8%)
>50 years	117 (54.2%)
Primary tumor histology	
Ductal	184 (85.2%)
Lobular	12 (5.6%)
Other	20 (9.3%)
Primary tumor grade	
I	22 (10.2%)
II	180 (83.3%)
III	14 (6.5%)
Hormonal receptor status	184 (85.2%)
Positive	143 (66.2%)
Negative	73 (33.8%)
HER2	
Positive	42 (19.4%)
Negative	174 (80.6%)
Ki67	14 (6.5%)
>14%	147 (68.1%)
≤14%	69 (31.9%)
AJCC stage	
I	76 (35.2%)
II	140 (64.8%)

AJCC: American joint committee on cancer.

### Lymph node status

SLNB using the methylene blue dye technique was successful in 99.1% of patients. A mean of 2.3 (range: 1–5) SLNs were excised, with 1 SLN in 54 patients, 2 SLNs in 78 patients, 3 SLNs in 60 patients, and >3 SLNs in 22 patients. A mean of 1.3 (range 1–2) SALN were identified by ultrasound and excised, including 1 SALN in 150 patients and 2 SALNs in 64 patients (Table 2). A mean of 20.3 (range: 12–36) lymph nodes were excised by ALND.

**Table 2:** **Presence of node metastases and pathologic features of the primary tumors**

Characteristic	Value
N of nodes excised, mean (range)	20.3 (12–36)
N of SLN excised, mean (range)	2.3 (1–5) (1 node in 54 cases, 2 nodes in 78 cases, 3 nodes in 60 cases, >3 nodes in 22 cases)
N of suspicious lymph node excised, mean (range)	1.3 (1–2) (1 node in 150 cases, 2 nodes in 64 cases)
Nodal metastases	
No metastasis	145 (67.1%)
Metastases	71 (32.9%)

SLN: sentinel lymph node.

Postoperative pathological examination of axillary lymph nodes obtained by ALND showed that 143 cases were negative, and that 71 cases were positive. Among these 71 positive cases, SLNB alone indicated that 63 were node-positive. Ultrasound indicated suspicious lymph nodes in 35 patients. Therefore, using the combination of SLNB and USALNB, 69 cases were node-positive.

### Sentinel lymph node biopsy alone

Table 3 shows the predictive value of SLNB alone. Using ALND results as the gold standard, sensitivity was 88.7%, specificity was 100%, false negative rate was 11.3% and false positive rate was 0%.

**Table 3:** **Comparison of the pathological results of SLNB between methylene blue and H&E staining**

SLNB	ALNB		Total
	+	-	
**+**	63	0	63
**-**	8	143	151
**Total**	71	143	214

Sensitivity = 63/71 = 88.7%.

Specificity = 143/143 = 100%.

False negative rate = 8/71 = 11.3%.

False positive rate = 0/143 = 0%.

SLNB: sentinel lymph node biopsy.

### Sentinel lymph node biopsy and ultrasound-suspicious lymph node biopsy

Table 4 shows the predictive value of SLNB + USALNB. Using ALND results as the gold standard, sensitivity was 97.2%, specificity was 100%, false negative rate was 2.8% and false positive rate was 0%.

**Table 4:** **Comparison, pathological results between methylene blue staining of SLNB + suspicious lymph node biopsy, pathological results**

SLNB + USALNB	ALNB		Total
	+	-	
**+**	69	0	69
**-**	2	143	145
**Total**	71	143	214

Sensitivity = 69/71 = 97.2%.

Specificity = 143/143 = 100%.

False negative rate = 2/71 = 2.8%.

False positive rate = 0/143 = 0%.

SLNB: sentinel lymph node biopsy.

Table 5 shows the predictive value of SLNB + USALNB. Using SLNB results as the gold standard, compared with SLNB alone, SLNB + USALNB had a better false negative rate (2.8% vs. 11.3%, P = 0.031).

**Table 5:** **Comparison of pathological results between SLNB and combined use of SLNB, suspicious lymph node biopsy**

SLNB	SLNB + USALNB		Total
	+	-	
**+**	63	0	63
**-**	6	145	151
**Total**	69	145	214

SLNB: sentinel lymph node biopsy.

Compared with SLNB, SLNB + USALNB had a better false negative rate (2.8% vs. 11.3%, P = 0.031).

### Follow-up data

During follow-up period (mean of 19 ± 4.5 months), only two patients were noted to develop distant metastatic disease.

The first patient was a 65-year-old female patient with grade II invasive ductal carcinoma (left breast, 4 cm). She underwent a level II ALND, and all sampled lymph nodes were negative. Tumor immunohistochemistry revealed a triple-negative breast cancer, P53-positive, and the Ki-67 index was <40%. She received four cycles of docetaxel and cyclophosphamide. Pulmonary and bone metastases were found one year after surgery.

The second patient was a 31-year-old female patient with grade II invasive ductal carcinoma (left breast, 1.7 cm). She underwent level II ALND and all sampled lymph nodes were negative. She received epirubicine, 5-fluorouracil and cyclophosphamide for 6 cycles, followed by 2 months of oral toremifene. The patient stopped her hormonal therapy by herself, and thoracic vertebral metastases were found two years after surgery.

## Discussion

Axillary lymph node status is one of the most important prognostic factors in patients with breast cancer, and is used for the selection of the appropriate adjuvant therapy. ALND was previously the standard management of the axilla in breast cancer, but ALND failed to add benefits to node-negative patients. In addition, ALND results in certain complications, such as upper limb edema and shoulder joint movement disorders. Recent studies using large randomized samples, such as the ALMANAC [14] and NSABP B-32 [15] trials, have shown that the false negative rate of the axillary lymph node status predicted by SLNB was 5-10%. False negatives affect the staging and proper choice of treatment.

Therefore, the aim of the present study was to assess the value of USALNB in patients with early breast cancer, and to compare SLNB + USALNB with SLNB alone. Results showed that SLNB + USALNB resulted in a significantly better false negative rate (2.8%) compared with SLNB alone (11.3%).

Preoperative ultrasound can be used to make a preliminary assessment of the status of the axillary lymph nodes [16,17]. Ultrasound is particularly important for patients with smaller non-palpable lymph nodes. Metastatic lymph nodes present some typical characteristics, such as enlargement, signal heterogeneity, and disorderly flow distribution [13]. However, in clinical practice, some metastatic lymph nodes show no typical manifestation. Based on their location and imaging findings, the most likely metastatic lymph nodes were regarded as the suspicious lymph nodes. Previous studies have shown that an overlap exists between the SLNs and the suspicious lymph nodes [18,19]. In the present study, some suspicious lymph nodes (82/214, 38.3%) were indeed SLNs. Thus, SLNB was easier to conduct, and the possibility of missing a positive lymph node was reduced.

The factors contributing the occurrence of false negative results during SLNB can be multifactorial, including variability in lymphatic drainage pathway, variability in SLNB techniques utilized, varying levels of surgeon experience, and patient selection issues. All current methodologies for SLNB, including the blue dye technique alone, the isotope technique alone, or the combined blue dye/isotope technique, rely on the lymphatic drainage pathways for successful outcome. Lymphatic drainage pathways depends on the integrity and physiology of the lymphatic channels and the corresponding lymph nodes [20]. Once the integrity of these structures is disrupted or altered, the lymphatic flow direction may also be disrupted or altered [21], which may in turn lead to false negative results. Investigations have suggested that the lymphatic drainage pathways for the nipple/areola complex region of the breast and other areas of the breast can geographically differ [22]. This difference may also contribute to variability in the degree of false negative results reported by various investigators [4,22-24]. Thus, the use of a combined approach of SLNB with ultrasound-guided axillary sampling of suspicious axillary lymph nodes could represent an advantageous alternative for the assessment of the axillary lymph node status. Previous studies on the use of four to five lymph node samplings from the axilla [25,26] showed a false negative rate similar to that of SLNB, despite ultrasound, magnetic resonance imaging, positron emission tomography and computed tomography. Furthermore, some studies showed that four lymph nodes may be dissected as a supplement to SLNB to reduce the false negative rate [27]. However, the results reported in the literature are inconsistent [28]. Numerous studies recently addressed ultrasound-guided axillary sampling as an alternate/additional staging procedure to improve the accuracy of SLNB [29,30]. However, the use of ultrasound alone to guide the biopsy of axillary lymph nodes (performed using either FNA or core needle biopsy) resulted in highly variable false negative rates [5], thus compromising its use in the clinical setting. Compared with the false negative rate of 11.3% using SLNB alone, the present study showed that ultrasound-guided axillary sampling of suspicious axillary lymph nodes along with SLNB yielded a significantly lower false negative rate of 2.8%. Thus, ultrasound-guided axillary sampling of suspicious axillary lymph nodes improved the accuracy of the staging of the axillary lymph nodes. This suggests that the addition of ultrasound-guided axillary sampling of suspicious axillary lymph nodes to axillary staging can effectively complement the standard utilization of SLNB alone, and for which the combined use of these two methodologies in axillary staging could have more far-reaching impact on determining more appropriate adjuvant therapies. However, the lack of a consensus on the standardization of quantitative indicators for defining suspicious axillary lymph nodes on ultrasound evaluation remains a significant issue, and contributes to the continued inability to effectively apply this methodology to clinical practice.

In many countries, SLNB is performed using an isotope (such as 99 m-Tc-colloid) in combination with a blue dye (such as methylene blue or isosulfan blue). However, in China, secondary to isotope shortage and governmental regulations, blue dye (i.e., methylene blue) is generally used alone for axillary staging for breast cancer, as in accordance with the breast cancer treatment guidelines and norms (2011 edition) from the Chinese Anti-Cancer Association. These established guidelines state that methylene blue may be used alone for SNLB, and that the results are reliable. Nevertheless, if isotope, such as 99 m-Tc-colloid, was more readily available to medical centers throughout China, then our current analysis would have been able to undertake a comparison of SLNB alone (by the methylene blue and isotope technique) versus SLNB (by the methylene blue and isotope technique) plus USALNB, which would be more applicable to the approach for axillary staging which is practiced internationally in other developed countries outside of China. Nevertheless, previous studies have demonstrated that the results from the use of a blue dye alone were comparable to the combined use of blue dye and isotope [31,32]. Furthermore, research has shown that the results from the use of methylene blue combined with an isotope were comparable to those obtained using isosulfan blue combined with an isotope [33]. Therefore, it is our belief that results of our present study regarding the role of adding USALNB to SLNB by blue dye alone (and without isotope) appear to be generalizable to the approach for axillary staging which is practiced internationally in other developed countries outside of China. However, realistically, prospective randomized clinical trials in larger patient populations will be necessary to confirm this.

The present study is not without limitations. First, the sample size was not very large, and the study was not randomized. In addition, the follow-up was short, and longer follow-up is necessary to correctly assess recurrences. Multicenter randomized studies should be performed to correctly assess the predictive value of SLNB + USALNB.

## Conclusion

The present study showed that USALNB + SLNB was better than SLNB alone. Moreover, this method was simple to use, and has potential clinical applications. However, the quantitative indicators of suspicious axillary lymph nodes need further investigation.
